# Transcranial Magnetic Resonance Imaging-Guided Focused Ultrasound with a 1.5 Tesla Scanner: A Prospective Intraindividual Comparison Study of Intraoperative Imaging

**DOI:** 10.3390/brainsci11010046

**Published:** 2021-01-04

**Authors:** Cesare Gagliardo, Roberto Cannella, Costanza D’Angelo, Patrizia Toia, Giuseppe Salvaggio, Paola Feraco, Maurizio Marrale, Domenico Gerardo Iacopino, Marco D’Amelio, Giuseppe La Tona, Ludovico La Grutta, Massimo Midiri

**Affiliations:** 1 Section of Radiological Sciences, Department of Biomedicine, Neuroscience and Advanced Diagnostics, University of Palermo, 90127 Palermo, Italy; rob.cannella89@gmail.com (R.C.); costanza.dangelo@gmail.com (C.D.); patrizia.toia@unipa.it (P.T.); p.salvaggio@libero.it (G.S.); giuseppe.latona@unipa.it (G.L.T.); ludovico.lagrutta@unipa.it (L.L.G.); massimo.midiri@unipa.it (M.M.); 2 Neuroradiology Unit, S. Chiara Hospital, 38122 Trento, Italy; paola.feraco@apss.tn.it; 3 Department of Physics and Chemistry, University of Palermo, 90133 Palermo, Italy; maurizio.marrale@unipa.it; 4 Section of Neurosurgery, Department of Biomedicine, Neuroscience and Advanced Diagnostics, University of Palermo, 90133 Palermo, Italy; gerardo.iacopino@unipa.it; 5 Section of Neurology, Department of Biomedicine, Neuroscience and Advanced Diagnostics, University of Palermo, 90133 Palermo, Italy; marco.damelio@unipa.it

**Keywords:** focused ultrasound, MR-guided focused ultrasound, high-intensity focused ultrasound ablation, magnetic resonance imaging, image quality, stereotaxic techniques, essential tremor

## Abstract

Background: High-quality intraoperative imaging is needed for optimal monitoring of patients undergoing transcranial MR-guided Focused Ultrasound (tcMRgFUS) thalamotomy. In this paper, we compare the intraoperative imaging obtained with dedicated FUS-Head coil and standard body radiofrequency coil in tcMRgFUS thalamotomy using 1.5-T MR scanner. Methods: This prospective study included adult patients undergoing tcMRgFUS for treatment of essential tremor. Intraoperative T2-weighted FRFSE sequences were acquired after the last high-energy sonication using a dedicated two-channel FUS-Head (2ch-FUS) coil and body radiofrequency (body-RF) coil. Postoperative follow-ups were performed at 48 h using an eight-channel phased-array (8ch-HEAD) coil. Two readers independently assessed the signal-to-noise ratio (SNR) and evaluated the presence of concentric lesional zones (zone I, II and III). Intraindividual differences in SNR and lesional findings were compared using the Wilcoxon signed rank sum test and McNemar test. Results: Eight patients underwent tcMRgFUS thalamotomy. Intraoperative T2-weighted FRFSE images acquired using the 2ch-FUS coil demonstrated significantly higher SNR (R1 median SNR: 10.54; R2: 9.52) compared to the body-RF coil (R1: 2.96, *p* < 0.001; R2: 2.99, *p* < 0.001). The SNR was lower compared to the 48-h follow-up (*p* < 0.001 for both readers). Intraoperative zone I and zone II were more commonly visualized using the 2ch-FUS coil (R1, *p* = 0.031 and *p* = 0.008, R2, *p* = 0.016, *p* = 0.008), without significant differences with 48-h follow-up (*p* ≥ 0.063). The inter-reader agreement was almost perfect for both SNR (ICC: 0.85) and lesional findings (*k*: 0.82–0.91). Conclusions: In the study population, the dedicated 2ch-FUS coil significantly improved the SNR and visualization of lesional zones on intraoperative imaging during tcMRgFUS performed with a 1.5-T MR scanner.

## 1. Introduction

Transcranial Magnetic Resonance Imaging-guided Focused Ultrasound (tcMRgFUS) is an emerging incisionless stereotactic procedure based on the thermal ablation of a brain area using a high-intensity focused ultrasound (HI-FU) beam. Randomized-controlled clinical trials and several prior studies have demonstrated the clinical efficacy of tcMRgFUS thalamotomy for the treatment of essential tremor [1,2,3], idiopathic asymmetrical tremor-dominant Parkinson’s disease [4,5], and neuropathic pain [6].

The treatment is conducted under constant Magnetic Resonance (MR) imaging monitoring. MR allows to acquire detailed anatomical images to calculate the optimal target coordinates and MR thermometry for real-time thermal monitoring during sonications [7]. Anatomical intraoperative MR images may also depict the typical neuroradiological findings of tcMRgFUS-placed lesions, which consist of three concentric lesional zones, as originally described by Wintermark et al. [8]. Optimal MR imaging quality is, therefore, of the upmost importance for the HI-FU thermal ablation and lesion monitoring. TcMRgFUS procedures have been initially performed using 3.0-T MR scanners, but this technology is also rapidly expanding on 1.5-T MR [7]. On 3.0-T MR scanners, the anatomical images and MR thermometry are typically acquired using standard body radiofrequency coil built in the MR system because the 30-cm-diameter hemispherical FUS helmet, stereotactic frame, and supporting equipment almost fill the whole scanner space and do not allow the placement of specific head coil [9]. When the tcMRgFUS equipment is integrated into a 1.5-T MR scanner, a dedicated coil is used to compensate the lower field strength. Initial studies have reported significant improvement in signal-to-noise ratio (SNR) using dedicated head coil on 1.5-T MR, but these evidences were only based on phantom evaluations [10]. Moreover, there are still very limited experiences on FUS thalamotomy performed with 1.5-T MR scanners [11,12,13]. Therefore, there is a significant gap in knowledge regarding the added value of dedicated coil on the quality of intraoperative imaging acquired in patients undergoing tcMRgFUS procedure with 1.5-T MR. We hypothesize that dedicated head coil significantly increases the image quality and intraoperative lesions detection in patients undergoing tcMRgFUS on 1.5-T MR, compared to the images acquired using the standard body radiofrequency coil.

The purpose of our study was to conduct a prospective intraindividual comparison between intraoperative sequences acquired using a dedicated head coil and a standard body radiofrequency coil in order to assess the signal-to-noise ratio and intraoperative visualization of neuroimaging findings in patients undergoing transcranial MR-guided Focused Ultrasound thalamotomy using 1.5-T MR.

## 2. Materials and Methods

The institutional review board approved this study (“Comitato Etico Palermo 1”—seduta del 11/04/2018 verbale n 04/2018). All subjects provided written informed consent before enrolling for tcMRgFUS treatment in accordance with the Declaration of Helsinki.

### 2.1. Patients

This prospective study included all the adult patients undergoing tcMRgFUS using a 1.5-T MR for the treatment of the movement disorders between September and December 2019. Candidates for tcMRgFUS were selected after accurate screening visits including extensive neurological evaluation and preprocedural CT and MR imaging for treatment planning. A total of 10 patients were treated during the study time. Two patients were excluded due to the onset of unbearable headache or nausea and vomiting during the treatment sonications, which made the patient unsuitable for performing additional sequences at the end of the procedure.

The following data were collected in the included patients: age, gender, diagnosis of movement disorder, skull density ratio (SDR) [14], and skull area obtained from preoperative evaluations. In addition, tcMRgFUS technical parameters were recorded from the dedicated treatment workstation (ExAblate, INSIGHTEC Ltd., Tirat Carmel, Haifa, Israel) after reviewing the procedures, including: total number of sonications, number of high-energy sonications (i.e., sonications reaching an average temperature > 50 °C), maximum and average temperatures, effective delivered energy (measured in Joules), sonication power (Watt), and duration (seconds) of each high-energy sonication.

### 2.2. Procedure Details

TcMRgFUS procedures were performed using a focused ultrasound (FUS) equipment (ExAblate 4000; InSightec Ltd., Tirat Carmel, Haifa, Israel) integrated with a 1.5-T MR unit (Signa HDxt; GE Medical Systems, Milwaukee, WI, USA). All treatments were performed by a single primary operator (C.G. with 15 years of experience in neuroradiology) who had the full control of the workstation, in collaboration with a dedicated transdisciplinary team.

Detailed descriptions of tcMRgFUS thalamotomy have been extensively described in prior reports [7,15]. Briefly, before the procedure, the patient’s head was comparatively shaved and immobilized to the FUS helmet using a stereotactic frame. A flexible silicone membrane, which integrates the 2-channel FUS-Head (2ch-FUS) coil, was placed on the patient’s head and the space between the head and the helmet was filled with cooled degassed water in order to allow the HI-FU transmission. Once the patient was positioned in the FUS table, 2D FRFSE T2-weighted sequences were acquired on coronal, sagittal, and axial planes according to the anterior commissure–posterior commissure (AC-PC) anatomical landmarks in order to calculate the optimal stereotactic coordinates. In our study, the target was placed in the nucleus ventralis intermedius (Vim) in the contralateral thalamus to the hand-dominant tremor side (25% of the AC-PC distance in front of PC, 2 mm above AC-PC line, and 11–12 mm lateral to the third ventricle wall).

The procedure began with the alignment stage (Stage I), which consisted of few low energy sonications (with a maximum temperature between 40 °C and 45 °C), to confirm the accuracy of thermal spot according to the frequency-encoding direction. During the verify stage (Stage II), multiple intermediate-energy sonications (with a maximum temperature of 50 °C which minimize the risk of a permanent lesion) were performed to evaluate the optimal treatment target based on real-time clinical assessment of transient tremor suppression or any type of adverse events. In case of poor tremor response, intraoperative anatomical images were used to redefine the target coordinates to achieve the optimal tremor suppression. The HI-FU ablation consisted of a few high-energy sonications, reaching an average temperature ≥ 51 °C (Stage III: treatment low) for permanent lesion and ≥55 °C (Stage IV: treatment high) for lesion consolidation. The number of high-energy sonications varied according to the patient’s skull characteristics, tremor disappearance, and neuroradiological findings on intraoperative imaging. Particularly, in our clinical practice, further high-energy sonications were usually considered for lesion consolidation in case of intraoperative imaging showing a FUS-placed lesion lacking of concentric lesional zones (zone I and zone II, see below).

### 2.3. MRI Data Acquisition

Intraoperative imaging was performed using a dedicated two-channel FUS-Head coil (INSIGHTEC Ltd., Tirat Carmel, Haifa, Israel). The coil is composed of two silicon-coated rings embedded in the elastic membrane. The two rings of this coil are positioned to either side of the patient’s head without additional mechanical constraints to the patient. For the purpose of this study, intraoperative axial 2D fast recovery fast spin echo (FRFSE) T2-weighed sequences were acquired immediately after the last high-energy sonication using the dedicated 2ch-FUS coil in all patients. Then, a second axial FRFSE T2-weighed sequence was scanned with the identical MR parameters and conditions, but it was acquired using the standard body radiofrequency (body-RF) coil integrated in the MR scanner. Both sequences were acquired with the same setup used during the procedure, and thus, before emptying the helmet from the coupling degassed cooled water. The same sequence, along with a specific MR brain protocol (axial 3D T1w BRAVO, sagittal 3D T2w FLAIR with fat saturation, axial 3D SWAN, axial 2D T2w FRFSE, axial 2D EPI-DWI, axial 2D T1 FSE; after i.v. contrast medium injection axial 3D T1w BRAVO and axial 2D T1 FSE), was repeated at the 48-h follow-ups using an eight-channel phased-array head (8ch-HEAD) coil (GE’s standard product 8ch BRAIN HD coil). Acquisition parameters for the sequences included in this study are reported in Table 1.

### 2.4. MRI Data Analysis

Two readers (R1 R.C. and R2 C.D., with 6 and 2 years of experience in neuroimaging) independently analyzed the intraoperative and postoperative axial FRFSE T2-weighed images in order to evaluate the SNR and presence of concentric lesional zones. All the images were analyzed using a dedicated workstation equipped with Horos (Annapolis, MD, USA) a free and open source code software program that is distributed free of charge under the Lesser General Public License (LGPL) at Horosproject.org. The sequences were anonymized and reviewed in random order.

**Figure 1:**
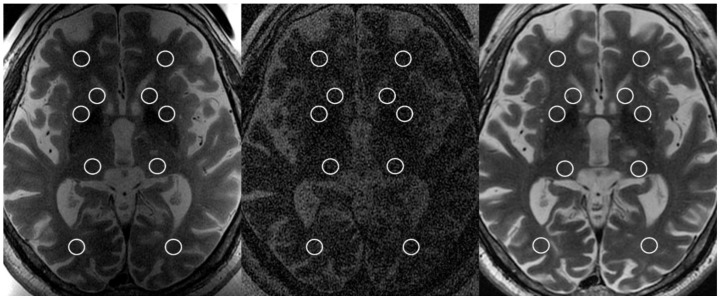
Axial T2-weighted FRFSE images acquired intraoperatively using the dedicated two-channel FUS-Head coil (**left image**); body radiofrequency coil (**central image**); and at 48-h using the eight-channel phased-array head coil (**right image**) at the level of the anterior commissure-posterior commissure plane, showing the placement of regions of interest for the evaluation of signal-to-noise ratio.

The SNR was evaluated following the approach proposed by the National Electrical Manufacturers Association (NEMA) [16]. A standard region of interest (ROI) with an area of 50 mm^2^ was placed on the axial FRFSE T2-weighed images at the level of the AP-PC plane (treatment plane) in each of the following locations, bilaterally (Figure 1): (a) white matter of the frontal lobe; (b) head of the caudate nucleus; (c) lentiform nucleus; (d) posterior aspect of the thalamus; and (e) white matter of the occipital lobe. ROIs were placed carefully avoiding lateral ventricles white matter and FUS-placed lesions and related imaging findings (i.e., vasogenic edema). Two additional ROIs were placed in the body of the lateral ventricles. The signal was recorded as the mean pixel value within the ROI, while the noise was defined as the variations (i.e., standard deviation) of pixel intensities. The SNR was then calculated using the following equation: SNR = (√(2 × S))/σ; where S is the mean signal in a ROI, and σ is the standard deviation from the same ROI.

The readers also recorded the presence of the three concentric lesional zones in each sequence, as described in prior studies [8,12]. Zone I was defined as central spot markedly hypointense on T2-weighted images, and it represents the cavitating lesion. Zone II was considered as moderate-to-markedly hyperintense area on T2-weighted images, concentrically surrounding the zone I, and demarcated by a hypointense rim, which represents the cytotoxic edema. Zone III consisted of a peripheral slightly hyperintense area, which represents the perilesional vasogenic edema surrounding the ablation lesion.

### 2.5. Statistical Analysis

Data were summarized as continuous variables and expressed as mean and standard deviation (SD) or median and interquartile range (IQR), and categorical variable, expressed as numbers and percentages. The Shapiro–Wilk test was performed to assess the normality distribution of continuous variables. Intraindividual differences in SNR between sequences acquired with different coils were compared using the Wilcoxon signed rank sum test. Differences in qualitative imaging analysis were assessed using the McNemar Test. The intraclass correlation coefficient (ICC), with 95% confidence intervals (95% CI), was calculated to assess the inter-reader agreement for continuous variables (SNR), while the Cohen’s kappa (*k*) test, with 95% confidence intervals (95% CI), was used for categorical variables. Agreement was categorized as poor (<0.00), slight (0.00–0.20), fair (0.21–0.40), moderate (0.41–0.60), substantial (0.61–0.80), or almost perfect (0.81–1.00). Statistical significance level was set at *p* < 0.05. Statistical analysis was conducted using SPSS software (Version 20.0. Armonk, NY, USA: IBM Corp).

## 3. Results

### 3.1. Patients

The characteristics of the final population and tcMRgFUS sonications parameters are summarized in Table 2. A total number of eight patients were protectively enrolled for the purpose of this study, including seven men and one woman with a mean age of 74.1 ± 5.4 years (range 65–81 years). All patients underwent tcMRgFUS for the treatment of essential tremor. All the TcMRgFUS thalamotomies were performed on the left Vim, according to the hand-dominant tremor side. The mean SDR was 0.48 ± 0.04 (range 0.42–0.56). The number of high-energy sonications reaching an average temperature greater than 50 °C ranged from two to five. The maximum temperatures in treatments sonications ranged from 51 to 62, while the average temperature ranged from 49 to 57.

### 3.2. Signal-to-Noise Ratio

A total number of 288 ROIs (12 ROIs in each sequence) were placed by each reader in order to assess the signal-to-noise ratio. The SNR on axial T2-weighted FRFSE acquired after the last high-energy sonication using different coils and at 48 h are reported in Table 3.

**Figure 2:**
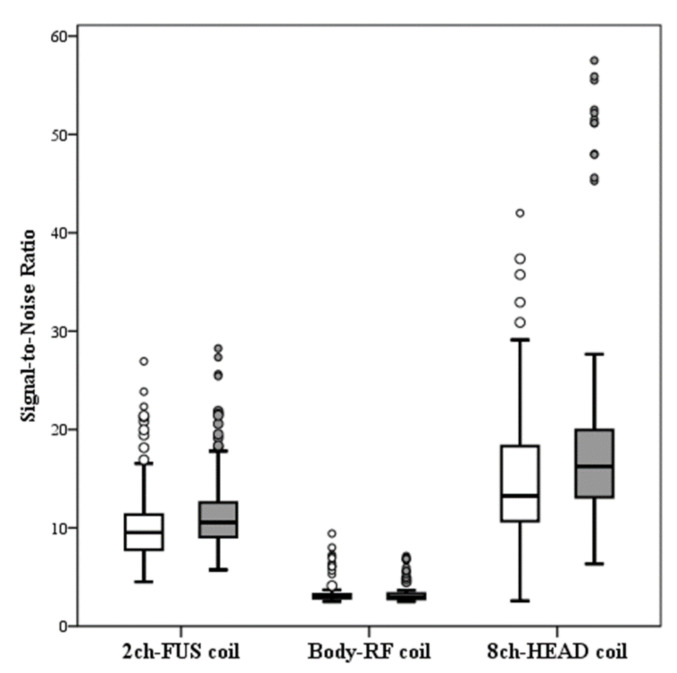
Plot box illustrates the distribution of signal-to-noise ratio in the images acquired using the dedicated two-channel FUS-Head (2ch-FUS) coil, body radiofrequency (body-RF) coil and eight-channel phased-array head (8ch-HEAD) coil by Reader 1 (white boxes) and Reader 2 (grey boxes).

Intraoperative axial T2-weighted FRFSE images acquired using the dedicated 2ch-FUS coil demonstrated a significantly higher SNR (R1, median SNR: 10.54, IQR: 9.05, 12.61; R2, median SNR: 9.52, IQR: 7.74, 11.36) compared to the images acquired with the body-RF coil (R1, median SNR: 2.96, IQR: 2.77, 3.31, *p* < 0.001; R2, median SNR: 2.99, IQR: 2.83, 3.26, *p* < 0.001) (Figure 2). The dedicated 2ch-FUS coil allowed to increase the SNR on intraoperative images by an average of 254 ± 74% and 211 ± 86% measured by R1 and R2, respectively. However, when compared with the standard 8ch-HEAD coil, the dedicated 2ch-FUS coil achieved significantly lower SNR (*p* < 0.001 for both readers), with a loss of SNR on intraoperative images of 31 ± 24% for R1 and 15 ± 46% for R2, compared to the images acquired at 48-h follow-ups.

The inter-reader agreement for SNR measurements was almost perfect (ICC: 0.85, 95% CI: 0.78, 0.89).

### 3.3. Qualitative Imaging Findings

The visualization rate of concentric lesional zones at intraoperative and 48-h imaging are reported in Table 4. On intraoperative axial 2D FRFSE T2-weighted images, zone I was already visible in 75% of patients for R1 and 100% for R2 in the images acquired with the dedicated 2ch-FUS coil, while it was never visualized when the images were acquired with the body-RF coil (*p* = 0.031 and *p* = 0.008 for R1 and R2, respectively). Similarly, zone II was always observed by both readers in the images acquired with the dedicated 2ch-FUS coil, while it was recorded only in one (12.5%) case by R1 (*p* = 0.016) and in no case by R2 (*p* = 0.008) on the subsequent acquisition performed with body-RF coil. There was no difference in the visualization rate of zone III on intraoperative images acquired by both coils (Table 4).

At 48-h follow-up, the three concentric zones were visualized in all patients by the two readers. There was no significant difference in the visualization of the three concentric zones between the intraoperative imaging acquired with dedicated 2ch-FUS coil and the 48 h follow-up, although zone III was more commonly visible at 48 h (37.5% vs. 100%, *p* = 0.063).

**Figure 3:**
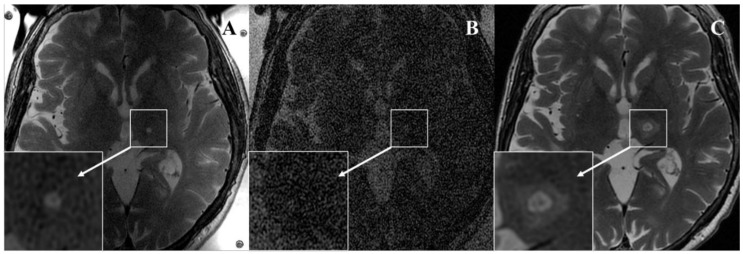
Seventy-eight-year-old woman with essential tremor. (**A**) Intraoperative axial 2D FRFSE T2-weighted sequence acquired after the last high-energy sonication using the dedicated two-channel FUS-Head coil well demonstrates the presence of zone I (hypointense) and zone II (hyperintense) in the left nucleus ventralis intermedius. (**B**) Intraoperative axial 2D FRFSE T2-weighted sequence acquired using body radiofrequency coil does not visualize any lesional zones. (**C**) Forty-eight-hour follow-up acquired with the eight-channel phased-array head coil demonstrates enlargement of zone I and zone II and development of peripheral vasogenic edema (zone III). Both readers agreed on the presence of these imaging findings.

The inter-reader agreement was almost perfect for all three concentric zones (zone I, *k*: 0.82; zone II, *k*: 0.90; zone III, *k*: 0.91). An example of T2-weighted FRFSE intraoperative images acquired after the last sonication using both coils and at 48-h follow-up is reported in Figure 3.

## 4. Discussion and Future Directions

In this study, intraoperative tcMRgFUS anatomical MR sequences were prospectively acquired after the last high-energy sonication using a dedicated 2ch-FUS coil and the standard body-RF coil on a 1.5-T MR scanner. Our results demonstrate that the dedicated 2ch-FUS coil significantly increased the SNR (*p* < 0.001) compared to the identical images acquired with the body-RF coil. Overall, the SNR increased by an average of 254 ± 74% for R1 and 211 ± 86% for R2 on axial T2-weighed FRFSE sequence. To the best of our knowledge, this is the first study evaluating the improvements of intraoperative neuroimaging quality acquired in patients undergoing tcMRgFUS with 1.5-T MR for the treatment of essential tremor. A recent technical note [10] reported that the 2ch-FUS coil allowed to increase the SNR by 10 times using the 1.5-T MR. However, those results were based only on measurements performed using a gel phantom [10]. Werner et al. [17] also described a custom-built eight-channel phased head receive array coil integrated to 3.0-T scanner. Their experience reports an increase of SNR by a factor of 3.5 times in intraoperative imaging compared to the standard body coil [17]. However, this preliminary report did not assess the intraoperative visualization of concentric lesional zones.

High quality imaging during tcMRgFUS is particularly challenging due to the presence of FUS equipment composed of a 30-cm-diameter water-filled helmet which fills almost the entire space within the MR scanner. As a consequence, the image quality with body-RF coil results in being inferior to the current neuroradiological diagnostic standards in terms of SNR and image artifacts, even when using 3.0-T scanners [9,17]. Despite the use of dedicated 2ch-FUS coil, a similar trend was observed in our study using 1.5-T MR scanner. The intraoperative SNR remained significantly lower compared to the images acquired at 48-h using the 8ch-HEAD coil (*p* < 0.001). However, there was only a mean SNR decrease of 31 ± 24% for R1 and 15 ± 46% for R2. This lower SNR did not impede the visualization of typical lesional zones on intraoperative imaging when using the 2ch-FUS coil and allowed us to schedule the first MRI follow-up at 48-h post-treatment.

In our study, the intraoperative images acquired after the last high-energy sonication using the 2ch-FUS coil allowed the optimal visualization of ablation zones (zone I and zone II) in almost all patients (75% by R1 and 100% by R2). Despite the fact that body-RF coil sequence was acquired after being scanned using the 2ch-FUS coil in all patients, giving potential time for further lesion consolidation, zone I was not visualized in any patient by R1 and R2 (*p* = 0.031 and *p* = 0.008, respectively), while zone II was scored in only one case by R1 and in no patient by R2 (*p* = 0.016 and *p* = 0.008, respectively). In prior studies, neuroimaging lesional findings were most commonly described in the MR images acquired immediately after the procedure or at 24-h follow-ups [8]. Intraoperative high-resolution images that accurately detect the lesional findings, without emptying the water-filled FUS helmet or removing the stereotactic frame, may have a significant impact for the treatments monitoring and early detection of any possible adverse events [12]. In our clinical experience, intraoperative axial FRFSE T2-weighed images are acquired between the high-energy sonications according to the real-time clinical evaluation and patients’ symptoms. Although the decision to stop the treatment is mainly based on the clinical efficacy and tremor suppression, the intraoperative visualization of the lesion is fundamental to document the correct location of the ablation zone and adequate lesion volume at the end of the treatment, without compromising the possibility of further sonications.

During follow-ups, the FUS-placed lesions typically enlarged in the first 48 h and then started to gradually decrease after 1 week [8,18,19]. In our study, all three concentric lesional zones were observed at 48 h in all the patients by both readers. As expected, zone III, representing the vasogenic edema, was more frequently observed at 48 h compared to intraoperative imaging (100% vs. 37.5%), although this difference did not reach the statistically significant level (*p* = 0.063 for both R1 and R2).

Our results may have significant implications for the further expansion of tcMRgFUS unique clinical and research applications. Optimization of high-quality intraoperative imaging may be necessary for making the tcMRgFUS a feasible image-guided intervention for the precise intracranial tumor ablation [20,21], target identification in psychiatric disorders [22,23,24,25], and reversible controlled blood-brain barrier opening to enhance therapeutic drugs delivery in specific brain regions [26,27,28,29,30], even using 1.5-T MR scanners. In this context, precise intraoperative imaging may provide accurate real-time delineation of pathological areas and direct visualization of the targeted spot, increasing the preciseness of the procedure and ensuring the preservation of nontarget tissues. All this will be even more interesting if other impulse sequences and/or weighting to be used in the intra-procedural phase will be implemented.

The most important limitation of our study is the small number of included patients. Performing additional time-consuming sequences (each with a scanning time of about four minutes) at the end of the procedure increases the total treatment time, which usually ranges from 2 to 3 h. However, despite the small population, there were already unequivocal differences in quantitative and qualitative analyses. Furthermore, our study did not compare the SNR and lesions visualization with the image quality achieved with 3.0-T MR unit using the standard body-RF coil. Further multicentric studies should be performed to compare the image quality and intraoperative neuroimaging findings in treatment performed with 1.5-T or 3.0-T MR scanners. Finally, we did not evaluate the correlations among intraoperative imaging findings, sonications parameters, patients’ characteristics, and clinical outcome at subsequent follow-ups, since other studies [9,31,32,33] have already assessed these aspects in larger cohorts.

## 5. Conclusions

In conclusion, the dedicated two-channel FUS-Head coil significantly increases the SNR on intraoperative anatomical images when the tcMRgFUS treatment is performed on 1.5-T MR scanner. We anticipate that the use of a dedicated coil with 3-T integrated tcMRgFUS systems is desirable and would fill the gap with nowadays 1.5-T integrated scanners. Anatomical high-resolution intraoperative images allow the accurate visualization of concentrically lesional zones after the high-energy sonications and may have a significant role in guiding the tcMRgFUS procedures. Considering that more and more often it is possible to use imaging techniques for the identification of specific biomarkers or therapeutic targets, in consideration of the increasing advances in the field of trans-cranial focused ultrasound, high resolution intraoperative imaging could be the ideal companion for increasingly tailored therapies.

## Figures and Tables

**Table 1:** MRI acquisition parameters used for axial fast recovery fast spin echo (FRFSE) T2-weighed images acquired intraoperatively and at 48-h follow-up with 1.5-T MR scanner.

	Axial FRFSE T2-Weighed Images		
	2ch-FUS Coil	Body-RF Coil	8ch-HEAD Coil
**Slice thickness** (mm)	2.0	2.0	2.0
**Slice gap**	0	0	0
**Number or slice**	19	19	19
**TR** (ms)	4461	4461	4380
**TE** (ms)	103	103	108
**Matrix**	384 × 288	384 × 288	320 × 288
**NEX**	2	2	5
**FOV** (cm)	22 × 22	22 × 22	24 × 24
**Acquisition time** (min)	4:06	4:06	4:06

Abbreviations: TR: Repetition Time; TE: Echo Time; NEX: number of excitations; FOV: Field of View.

**Table 2:** Characteristics of the final treated population.

Characteristics	Number
**Patients**	8
**Sex**	
Males	7 (87.5%)
Females	1 (12.5%)
**Age** (years)	
Mean ± SD (range)	74.1 ± 5.4 (65–81)
**Thalamotomy side**	
Left Vim	8 (100%)
Right Vim	0 (0%)
**SDR**	
Mean ± SD (range)	0.48 ± 0.04 (0.42–0.59)
**Skull area**	
Mean ± SD (range)	343.6 ± 18.2 (323–371)
**Treatment elements**	
Mean ± SD (range)	948 ± 48.1 (839–996)
**Number of sonications**	
Mean ± SD (range)	11.7 ± 2.1 (9–15)
**Number of High-energy sonications** (Stage IV)	
Mean ± SD (range)	4.0 ± 1.0 (2–5)
**Energy** (Joule)	
Mean ± SD (range)	11696.5 ± 6646.4 (4536–28062)
**Power** (Watt)	
Mean ± SD (range)	628.7 ± 97.6 (438–791)
**Time** (seconds)	
Mean ± SD (range)	19.5 ± 8.5 (11–41)
**Maximum temperatures** (°C)	
Mean ± SD (range)	55.5 ± 2.7 (51–62)
**Average temperatures** (°C)	
Mean ± SD (range)	52.7 ± 2.3 (49–57)

Continuous variables are expressed as mean ± standard deviation (SD), categorical variables are expressed as numbers and percentages. Abbreviation: SDR: skull-density ratio.

**Table 3:** Signal-to-nose ratio (SNR) differences between coils.

	2ch-FUS SNR	Body-RF SNR	8ch-HEAD SNR	*p* Value 2ch-FUS vs. body-RF	*p* Value 2ch-FUS vs. 8ch-HEAD	ICC (95% CI)
**Reader 1**	10.54 (9.05, 12.61)	2.96 (2.77, 3.31)	16.24 (13.10, 19.95)	<0.001	<0.001	0.85 (0.78, 0.89)
**Reader 2**	9.52 (7.74, 11.36)	2.99 (2.83, 3.26)	13.24 (10.67, 18.31)	<0.001	<0.001	

Variables are expressed as median and interquartile range (25th to 75th percentile). Variables were compared using the Wilcoxon signed rank sum test. Inter-reader agreement was assessed using the intraclass correlation coefficient (ICC), with 95% confidence intervals (95% CI). Statistically significant values (*p* < 0.05) are highlighted in bold. Abbreviations: 2ch-FUS: Two-channel FUS-Head Coil; Body-RF: Body Radiofrequency Coil; 8ch-HEAD: Eight-channel phased-array head Coil; SNR: Signal-to-Noise Ratio; ICC: Intraclass Correlation Coefficient.

**Table 4:** Comparison of visualization of the three concentric zones on intraoperative images obtained with head and body coils.

	2ch-FUS	Body-RF	8ch-HEAD	*p* Value 2ch-FUS vs Body-RF	*p* Value 2ch-FUS vs 8ch-HEAD	*k* Value (95% CI)
**Zone I**						
Present						0.82 (0.59, 1.00)
Reader 1	6 (75.0)	0 (0)	8 (100)	**0.031**	0.500	
Reader 2	8 (100)	0 (0)	8 (100)	**0.008**	1.000	
**Zone II**						
Present						0.90 (0.71, 1.00)
Reader 1	8 (100)	1 (12.5)	8 (100)	**0.016**	1.00	
Reader 2	8 (100)	0 (0)	8 (100)	**0.008**	1.00	
**Zone III**						
Present						0.91 (0.75, 1.00)
Reader 1	3 (37.5)	2 (25.0)	8 (100)	1.000	0.063	
Reader 2	3 (37.5)	3 (37.5)	8 (100)	1.000	0.063	

Categorical variables (Zone I, II, III) are expressed as numbers and percentages in parenthesis and they were compared using the McNemar Test. Inter-reader agreement was assessed using the Cohen’s kappa (*k*) test with 95% confidence intervals (95% CI). Statistically significant values (*p* < 0.05) are highlighted in bold. Abbreviations: 2ch-FUS: Two-channel FUS-Head Coil; Body-RF: Body Radiofrequency Coil; 8ch-HEAD: Eight-channel phased-array head Coil.

## Data Availability

The datasets generated during and/or analysed during the current study are available from the corresponding author on reasonable request.
